# The histone chaperone DAXX maintains the structural organization of heterochromatin domains

**DOI:** 10.1186/s13072-015-0036-2

**Published:** 2015-10-21

**Authors:** Lindsy M. Rapkin, Kashif Ahmed, Stanimir Dulev, Ren Li, Hiroshi Kimura, Alexander M. Ishov, David P. Bazett-Jones

**Affiliations:** Program in Genetics and Genome Biology, The Hospital for Sick Children, Toronto, ON M5G 0A4 Canada; Department of Biochemistry, The University of Toronto, Toronto, ON M5S 1A8 Canada; Graduate School of Bioscience and Biotechnology, Tokyo Institute of Technology, 4259 Nagatsuta, Midori-Ku, Yokohama 226-8501 Japan; Department of Anatomy and Cell Biology, University of Florida College of Medicine, and University of Florida Cancer Center, Gainesville, FL 32610 USA

**Keywords:** Nuclear organization, Heterochromatin, Chromocentres, DAXX, Electron spectroscopic imaging

## Abstract

**Background:**

The death domain-associated protein (DAXX) collaborates with accessory proteins to deposit the histone variant H3.3 into mouse telomeric and pericentromeric repeat DNA. Pericentromeric repeats are the main genetic contributor to spatially discrete, compact, constitutive heterochromatic structures called chromocentres. Chromocentres are enriched in the H3K9me3 histone modification and serve as integral, functionally important components of nuclear organization. To date, the role of DAXX as an H3.3-specific histone chaperone has been investigated primarily using biochemical approaches which provide genome-wide views on cell populations and information on changes in local chromatin structures. However, the global chromatin and subnuclear reorganization events that coincide with these changes remain to be investigated.

**Results:**

Using electron spectroscopic imagine (ESI), a specialized form of energy-filtered transmission electron microscopy that allows us to visualize chromatin domains in situ with high contrast and spatial resolution, we show that in the absence of DAXX, H3K9me3-enriched domains are structurally altered and become uncoupled from major satellite DNA. In addition, the structural integrity of nucleoli and the organization of ribosomal DNA (rDNA) are disrupted. Moreover, the absence of DAXX leads to chromatin that is more sensitive, on a global level, to micrococcal nuclease digestion.

**Conclusions:**

We identify a novel role of DAXX as a major regulator of subnuclear organization through the maintenance of the global heterochromatin structural landscape. As well, we show, for the first time, that the loss of a histone chaperone can have severe consequences for global nuclear organization.

**Electronic supplementary material:**

The online version of this article (doi:10.1186/s13072-015-0036-2) contains supplementary material, which is available to authorized users.

## Background

An essential component of gene regulation is the organization of the eukaryotic genome into functionally and spatially discrete chromatin domains. These physical domains separate active, open regions of the genome from repressed, closed domains forming structural and molecular boundaries. These boundaries help shape the nucleus into microenvironments that not only control the activity of domain-associated regions of the genome, but guide the formation of subnuclear compartments and structures required for cellular function [12]. Therefore, identifying key factors involved in the establishment and maintenance of chromatin domains will provide critical insight into how chromatin landscapes regulate vital cellular processes, including those implicated in various diseases such as laminopathies and cancers where global chromatin architecture is disrupted [12, 55, 59].

Based on cytogenetic observations, there are two main classifications of chromatin: lightly staining euchromatin which undergoes cell-cycle-dependent stages of condensation and de-condensation, and the more densely stained heterochromatin that remains compact throughout most of the cell cycle [35]. In contrast to active or open euchromatin, heterochromatin forms at repressed regions of the genome and is currently grouped into either developmentally regulated facultative heterochromatin or the more static, constitutive heterochromatin domains consisting of telomeres and centric and pericentric loci [52, 56]. In mouse cells, pericentric constitutive heterochromatin domains form 8–30 chromocentres, which contain clusters of the AT-rich, 234-bp major satellite repeat contributed from multiple chromosomes [28, 60]. The resulting spatially discrete structures provide a scaffold for centromere function, contribute to sister chromatids cohesion, and are proposed to act as platforms to concentrate and control heterochromatic environments [4, 5, 28].

Heterochromatic regions of the genome are enriched in distinct sets of histone tail modifications, which include the methylation on H3K9, H3K27, and H4K20, and thus correlate with repressed regions of the genome [39]. Despite being preferentially associated with transcriptionally active genes, the replication-independent H3 variant H3.3 is required for the formation of heterochromatin in the developing mouse embryo [1, 53], and its deposition was observed in telomeric, pericentric repeats, and a subset of endogenous retroviral elements [19, 21, 25]. Whereas H3.3 is specifically deposited into active regions of the genome by the chaperone histone regulator A (HIRA), the complex containing the chromatin remodeler alpha thalassemia/mental retardation syndrome X-linked (ATRX) and the chaperone death domain-associated protein (DAXX) is responsible for H3.3 deposition into heterochromatin [19, 25, 51].

Although mechanistic details have yet to be elucidated, the involvement of the DAXX/ATRX chromatin assembly pathway in telomere stability and specificity for repetitive DNA elements, together with the requirement for H3.3 in the formation of heterochromatin in the developing mouse embryo, implicate DAXX as a key factor in heterochromatin maintenance [34, 42, 53]. Unlike telomeric and pericentric repeats, centromeric chromatin further harbors the histone H3 variant CenH3 [4].To date, these roles have been investigated primarily using biochemical approaches which provide genome-wide views on cell populations and information on changes in local chromatin structures [17, 19, 25]. Their limitations, however, are that global chromatin and subnuclear reorganization events that may precede these local changes cannot be addressed. Our approach, therefore, is to combine light microscopy (LM) with electron spectroscopic imaging (ESI), a specialized form of energy-filtered transmission electron microscopy, to visualize chromatin domains in situ with high contrast and spatial resolution [18], as a means of exploring the role of DAXX as a key regulator in the structural organization of global heterochromatin landscapes.

Here, we show that in the absence of DAXX, the repressive histone modification H3K9me3 no longer correlates with chromatin compaction, and aberrant associations between H3K9me3-enriched chromatin and both H3.3 and major satellite repeats are observed. Additionally, we found that DAXX-dependent heterochromatin organization is required to maintain the structural integrity of the nucleolus. Our results indicate that DAXX is required to maintain the structural organization of heterochromatin domains, and we show, for the first time, that the loss of a histone chaperone can have severe consequences for global nuclear organization and chromatin sensitivity.

## Results

### H3.3 is enriched in a fraction of H3K9me3-enriched domains

**Fig. 1:**
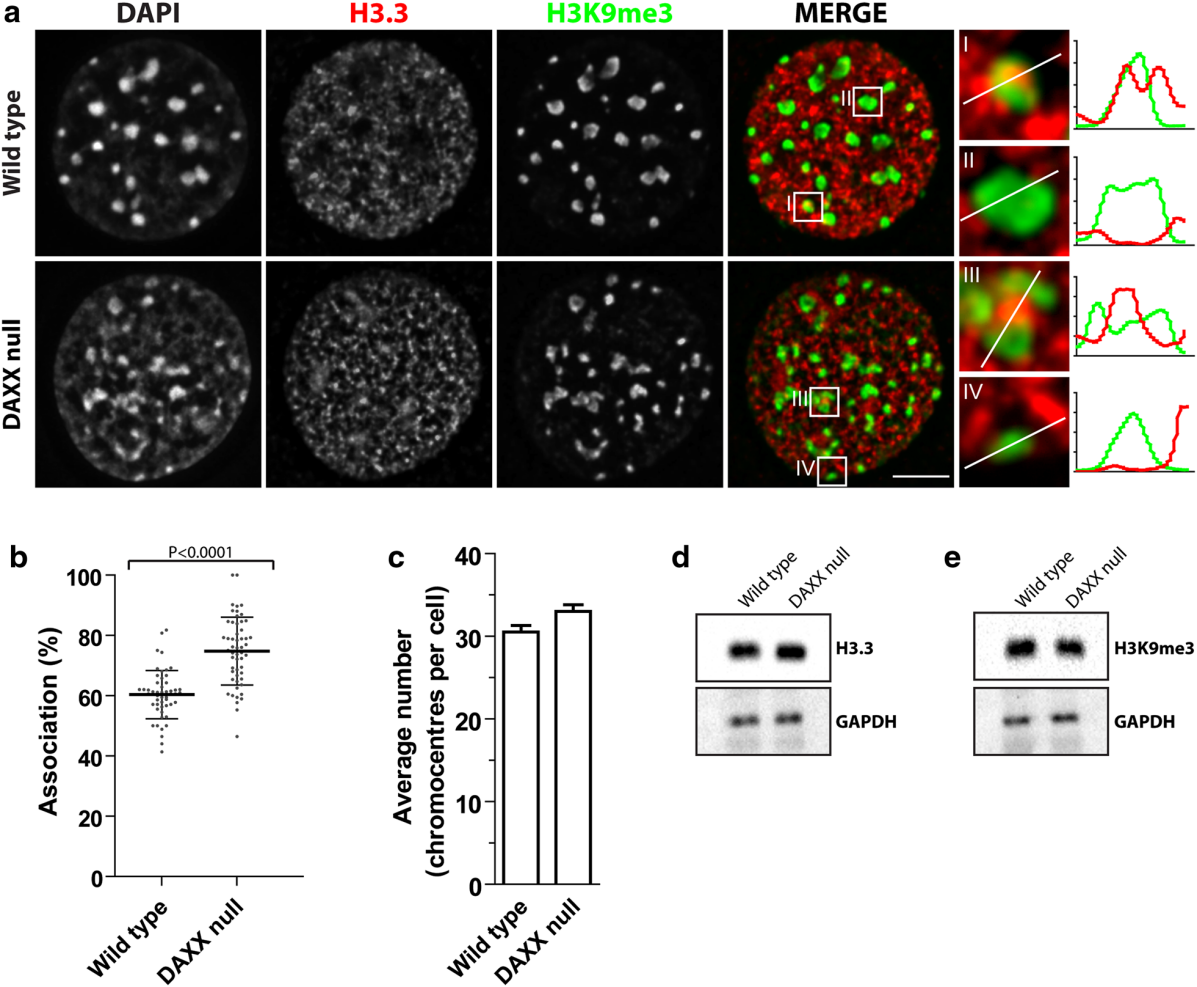
The association of H3.3 with H3K9me3-enriched chromatin is increased in the absence of DAXX. **a** Wild type and DAXX null fibroblasts immunolabeled for H3.3 (*red*) and H3K9me3 (*green*). Line scan intensity plots of H3.3 and H3K9me3 for magnified chromocentres, H3.3 associated (*I*, *III*) and non-associated (*II*, *IV*) chromocentres. *Scale bar* 5 µm. **b** Quantification of the percent association of H3.3 and H3K9me3-enriched chromatin domains in wild type (*n* = 52) and DAXX null (*n* = 54) cells. Data calculated as the mean percent of association per cell. **c** Quantification of the average number of chromocentres (H3K9me3-enriched foci). *Error bars* represent SEM from three independent experiments. **d**, **e** Western blot analysis of whole-cell lysates of H3.3 (**d**) and H3K9me3 (**e**)

The discovery of DAXX as an H3.3-specific chaperone responsible for its deposition in telomeres and pericentric heterochromatin [19, 25] indicated that H3.3 was not restricted to active regions of the genome. To determine the DAXX-dependent nuclear localization of endogenous H3.3, we examined wild type and DAXX null MEFs using immunofluorescence microscopy (Additional file 1). We observed that, within the resolution limit of fluorescence microscopy, H3.3 correlated strongly with the periphery of a fraction of H3K9me3-enriched domains but not significantly with others (Fig. 1a). Line scan intensity plots show H3.3-associated and non-associated H3K9me3-enriched domains from wild type and DAXX null cells. In this context, association is defined as overlap of the H3K9me3 and H3.3 fluorescence signals. The percent of H3K9me3-enriched domains associated with H3.3 is slightly greater in DAXX null cells with a mean of 74.8 (SE 1.5), yet only 60.4 (SE 1.1) in the control wild-type cells (Fig. 1b). This is not due to changes in the total number of chromocentres as the average number per nucleus are similar in both cell lines, with means of 30.5 (SE 5.1) in wild type and 33.0 (SE 5.1) in DAXX null cells (Fig. 1c). Western blot analysis of whole-cell lysates (Fig. 1d, e) showed that the expression levels of H3.3 and H3K9me3 are similar in both cells lines. As a control for epitope accessibility within compact chromatin domains, wild type and DAXX null cells were transiently transfected with a FLAG-H3.3 plasmid and immunolabeled with anti-FLAG primary antibodies (Additional file 2A). The sub-nuclear distributions of both endogenous and over-expressed H3.3 were equivalent, and line scan intensity plots again revealed that FLAG-H3.3 remained peripherally associated with H3K9me3-enriched domains; only a fraction of chromocentres were associated with FLAG-H3.3. The forced expression of H3.3 is thus not sufficient to drive H3.3 into the core of constitutive heterochromatin domains, but rather remains peripherally associated with H3K9me3-enriched regions. The peripheral association of H3.3 with H3K9me3-enriched domains was confirmed using correlative LM/ESI and immunogold labeling of H3.3 (Additional file 2B). We did not observe H3.3 in the core of the compact chromatin domains, rather it was found at the periphery of the chromocentres and in the euchromatic space.

### DAXX maintains the structural integrity of pericentric heterochromatin domains

**Fig. 2:**
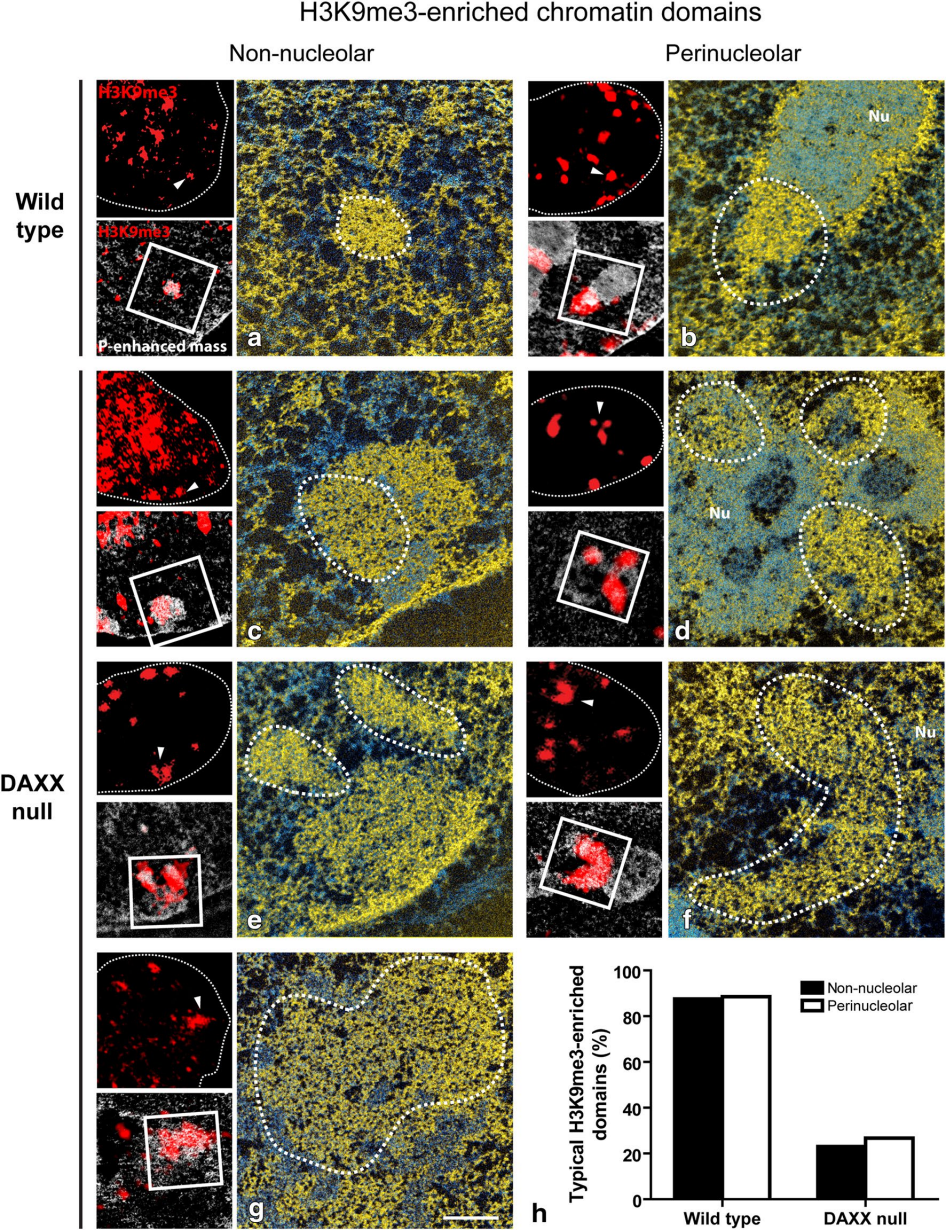
Disruption of chromocentres in the absence of DAXX. Correlative LM-ESI micrographs. H3K9me3 fluorescence (*left panel*, *top*) overlaid on the mass sensitive image generating the correlative image (*left panel*, *bottom*). High magnification ESI micrograph of the region shown in the *white box* (correlative image) of the corresponding H3K9me3-containing structure (*arrowhead*). Approximate boundaries of the H3K9me3 region are indicated by a *dashed line*. **a** Non-nucleolar and **b** perinucleolar heterochromatin domain of wild-type cell. **c**–**g** H3K9me3 correlative LM-ESI micrographs of DAXX null cells. (**c**, **e**, and **g**) Non-nucleolar and (**d** and **f**) perinucleolar heterochromatin structures. In all ESI images, chromatin is represented by levels of yellow and protein-based structures as cyan. *Nu* nucleolus. *Scale bar* 0.5 µm. **h** Quantification of the number of typical non-nucleolar and perinucleolar H3K9me3-containing heterochromatin domains in wild type and DAXX null cells

We next sought to determine if the significant increase in association of H3.3 with the periphery of H3K9me3-enriched domains observed in the absence of DAXX (Fig. 1a, b) affected the underlying chromatin structure of chromocentres. Therefore, wild type and DAXX null MEFS were prepared for correlative LM/ESI with antibodies specific to the H3K9me3 histone modification to mark pericentric constitutive heterochromatin domains. Physical sections on EM grids were imaged for fluorescence microscopy (Fig. 2a, top left panel) prior to imaging by ESI. The H3K9me3-positive regions were identified by correlation to the fluorescence image (Fig. 2a, bottom left panel) and imaged (Fig. 2a, arrowhead and white square). ESI micrographs of the H3K9me3-enriched domain were obtained and the approximate boundaries of the H3K9me3 signal outlined by dashed lines (Fig. 2a). The process was repeated for a total of 74 H3K9me3-enriched regions from wild type and 95 such regions from DAXX null cells. Since some centric and pericentric regions of the genome can be found adjacent to the nucleolus [11, 28], the H3K9me3-enriched regions were classified as either non-nucleolar or perinucleolar (nucleolar-associated) heterochromatin.

Consistent with previous reports, typical chromocentres from wild-type cells are radially symmetric structures that are comprised of compact chromatin, occupy discrete regions within the nucleus, and are readily discernible from the surrounding euchromatin (Fig. 2a) [20, 23]. Furthermore, the boundaries of the chromocentres, as approximated by the H3K9me3 fluorescence signal, correlate strongly with the regions of compact chromatin. A similar correlation between H3K9me3-enrichment and compact chromatin can be observed in perinucleolar heterochromatin domains (Fig. 2b). Importantly, despite the close spatial associations between H3K9me3-enriched chromatin and the nucleolus (Nu), the two regions form discrete structures. What should be appreciated in Fig. 2b is the clear “respect” for spatial boundaries, where both structures occupy discrete regions, albeit immediately juxtaposed. As such, the following four characteristics were used as criteria to classify non-nucleolar and perinucleolar-associated (adjacent) heterochromatin domains as either typical or atypical (1) compact chromatin (2) radial symmetry, (3) enriched in H3K9me3, and (4) sharp boundaries with other nuclear structures (i.e., surrounding euchromatin and nucleoli). Regions that met all four criteria were classified as typical, and those that did not, atypical. Mitotic cells, including those in early prophase were excluded from the analysis. With either fluorescence microscopy or ESI it is very easy to identify a cell in early prophase (and metaphase) by virtue of the chromatin/chromosome morphology. Therefore, the compaction observed through the loss of DAXX is not related to the chromosome condensation associated with mitosis.

We found the majority of non-nucleolar (87.5 %) and perinucleolar (88.5 %) chromocentres from wild-type cells were typical in nature (Fig. 2a, b). The H3K9me3-enriched regions (Fig. 2a, b; dashed lines) correlated with compact chromatin and were discernible from both the surrounding euchromatin and from the nucleolus. In contrast, only 23.0 % of non-nucleolar and 26.7 % of perinucleolar chromocentres from DAXX null cells were typical (Fig. 2c–g). Figure 2 shows representative images of the range of chromatin-related phenotypes observed in non-nucleolar (c, e, and g) and perinucleolar (d and e) chromocentres caused by the loss of DAXX. The chromocentre shown in Fig. 2c contains levels of non-chromatin protein-based structures not typically observed in chromocentres, even though the radial nature of the structure was retained. In this example, the H3K9me3 signal does not span the entire area of compaction of the non-nucleolar domain. Similarly, a loss of spatial correlation between H3K9me3-enrichment and compact chromatin can be seen in the non-nucleolar chromocentre of Fig. 2e. In this example, the H3K9me3 signal shows two regions of enrichment (arrowhead, dashed lines), yet a third connected domain of compact chromatin can be discerned that is depleted in the mark. Despite the absence of H3K9me3 in this region, the domain contains compact chromatin and can be readily distinguished from the surrounding chromatin. The example in Fig. 2g shows another phenotype characterized by large regions of compact chromatin interspersed with protein-based structures and compact chromatin that is no longer restricted to H3K9me3 domains; no morphologically distinct boundaries exist between the marked and unmarked compact chromatin.

We also observed a loss of spatial boundaries between perinucleolar heterochromatin and the nucleolus in the absence of DAXX (Fig. 2d, f). For example, in Fig. 2d, not only do the three regions of H3K9me3 signal (dashed lines) correlate with ranges of chromatin compaction, but the distinction between the nucleolus and the surrounding heterochromatin is lost. The chromocentre shown in Fig. 2f is found between two nucleoli. Although compact chromatin domains containing H3K9me3 labeling are observed, they contact a compact chromatin domain that does not correlate with the H3K9me3 modification. The two domains of compact chromatin, one containing H3K9me3 and one not, can be distinguished morphologically in the phosphorus distribution image. Moreover, the discrete boundary between compact chromatin and the nucleolus is lost. A quantification of the percentage of typical non-nucleolar and perinucleolar H3K9me3-enriched chromatin domains in the control and DAXX null cells is shown in Fig. 2h.

The observed phenomena are not cell-cycle dependent as different phenotypic classes were sometimes observed in a single cell. We conclude that in the absence of DAXX, the compact chromatin-containing chromocentres, both non-nucleolar and perinucleolar, are no longer restricted to H3K9me3 boundaries. As well, the spatial boundaries between perinucleolar heterochromatin domains and the nucleolus are severely compromised.

### Aberrant spatial relationships of H3K9me3-enriched chromatin and major satellite DNA

We further sought to determine whether H3K9me3-enriched chromatin domains that form in the absence of DAXX (Fig. 2) maintained the typical signatures of constitutive heterochromatin. Wild type and DAXX null cells were immunolabeled with antibodies specific to the repressive histone modifications H3K9me3 and H4K20me3 and the heterochromatin-associated protein HP1 [6, 10, 40]. We found that chromocentres from both cell lines are enriched in H3K9me3, H4K20me3, and HP1 (Additional file 3A, B).

**Fig. 3:**
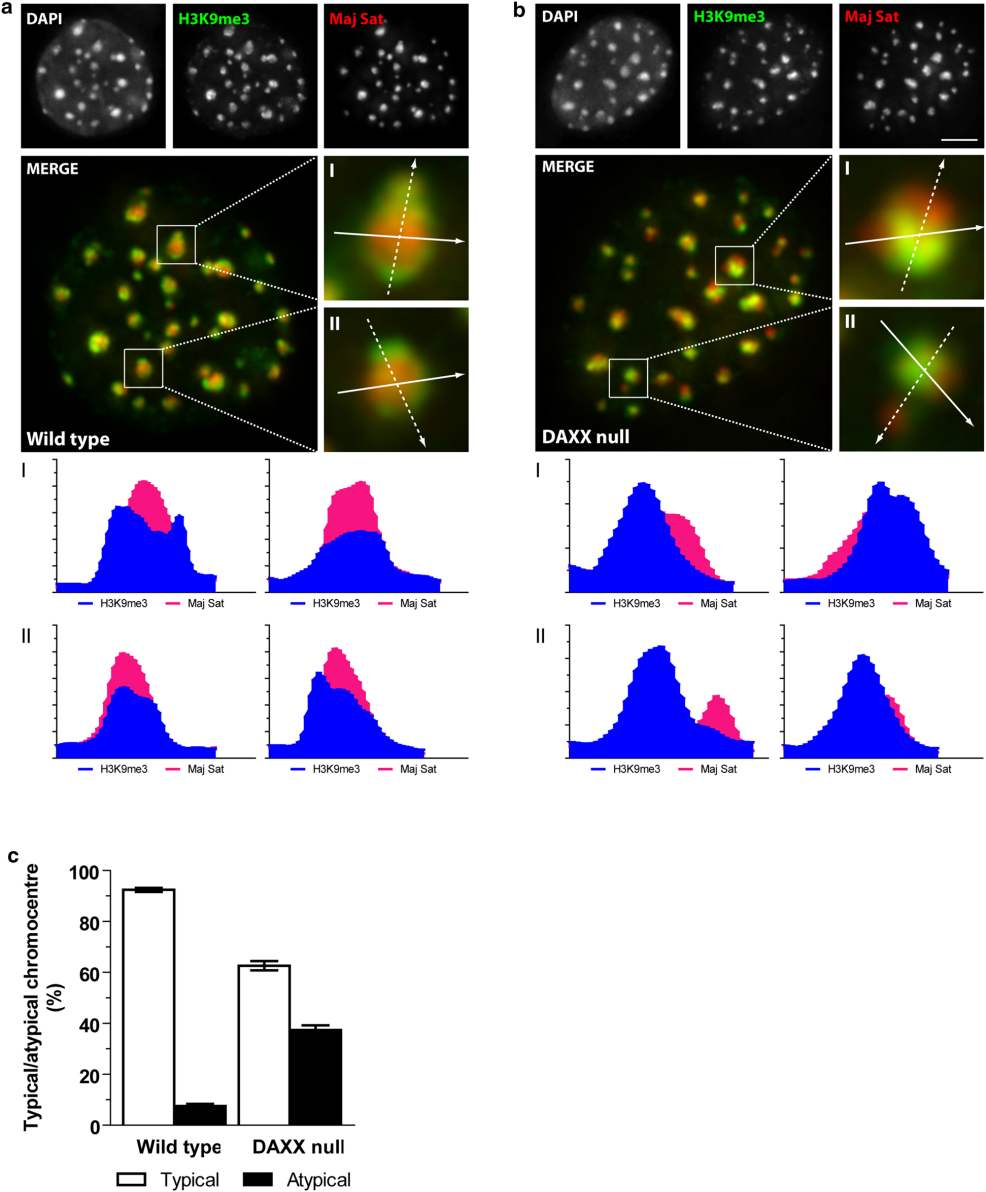
Aberrant spatial relationships between H3K9me3 and major satellite DNA. FISH of major satellite DNA (*red*) and immunofluorescence (IF) microscopy of H3K9me3 (*green*) of wild type (**a**) and DAXX null (**b**) fibroblasts. Magnified chromocentres are marked by *white boxes* in the merged images. Two independent line scan intensity plots are shown for each enlarged chromocentre. *Dashed solid arrows* indicate the line scan plot and direction of the first (*left*) and second (*right*) histograms, respectively. *Scale bar* 5 µm. **c** Quantification of the percentage of typical versus atypical H3K9me3-enriched chromatin domains from each cell line. *Error bars* SEM

Given the enrichment of major satellite DNA in chromocentres [28], we determined whether that association was conserved in the absence of DAXX. Using antibodies against H3K9me3 and DNA FISH probes against major satellite DNA, and consistent with previous reports [28], we observed that chromocentres from wild-type cells contain a core of major satellite repeat DNA packaged as H3K9me3-enriched chromatin (Fig. 3a). Line scan analysis of representative chromocentres demonstrates strong correlations in intensity peak profiles between the H3K9me3 and major satellite signals. Independent of the direction of the line scan, the major satellite signal is contained with radial symmetry in relation to the H3K9me3 area. In DAXX null cells, however, while approximately half of the chromocentres display the typical spatial relationship between H3K9me3 and major satellite DNA observed in control cells, approximately 50 % display aberrant spatial relationships (Fig. 3b). In these regions, major satellite DNA peak profiles are no longer centered on the core of the H3K9me3-defined region but extend asymmetrically beyond the H3K9me3 distribution. The mean percentage of the typical chromocentres in DAXX nulls (centrally located major satellite DNA) is 63.5 (SE 10.3, *n* = 1301) compared to wild-type cells where 92.4 (SE 7.6, *n* = 1222) chromocentres display typical H3K9me3 and major satellite spatial relationships (Fig. 3c). We conclude that pericentric satellite repeat DNA becomes uncoupled from its association with the H3K9me3 histone mark when DAXX is absent. Taken together, these data led us to conclude that DAXX plays a role in maintaining spatial relationships between compact chromatin, compact chromatin biochemically marked as constitutive heterochromatin by H3K9me3, and major satellite repeat DNA.

### Increased number of cells containing mini nucleoli

**Fig. 4:**
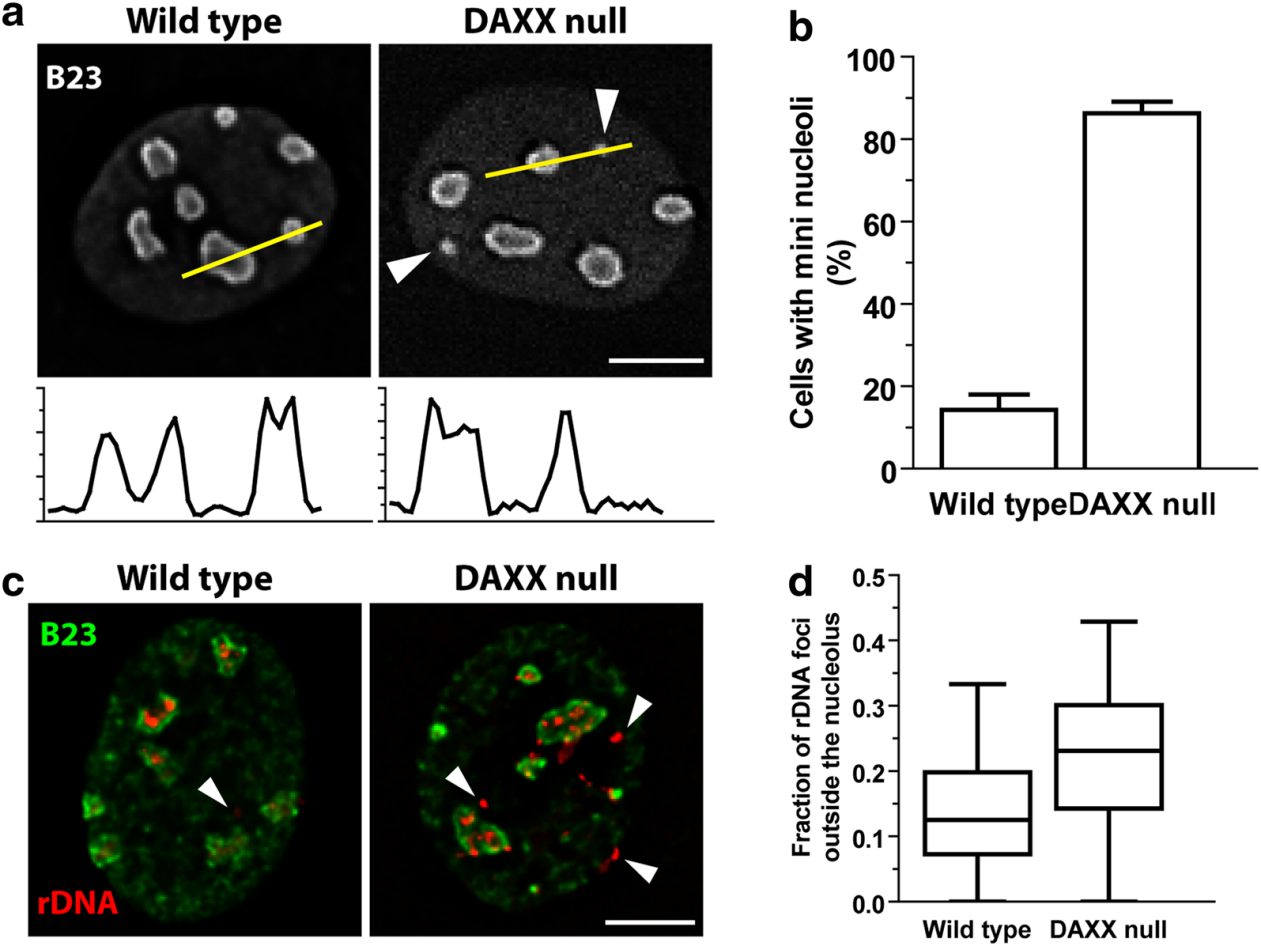
DAXX maintains the structural integrity of nucleoli and the organization of rDNA. **a** B23-labeled IF images. *Arrowheads* indicate the mini nucleoli. *Arrows* indicate the line scan intensity plot and direction. *Scale bar* 5 µm. **b** Quantification of the percentage of cells containing a minimum of one mini nucleolus. *Error bars* represent SEM of a minimum of 100 cells. **c** FISH of rDNA (*red*) and IF microscopy of B23 (*green*). *Arrowheads* indicate rDNA foci found outside of the B23-defined nucleolar boundaries. *Scale bar*, 5 µm. **d** Box plot of the fraction of rDNA foci found outside of the nucleolar boundaries

A structural relationship exists between centric and pericentric heterochromatin and the nucleolus [11, 28, 29, 49]. In Su(var)3–9 mutants that lack H3K9me chromatin, the cells displayed disorganized nucleoli [49]. We therefore wanted to determine if the observed disruptions in the organization of heterochromatin in the absence of DAXX, including the frequently observed loss of a discrete boundary between perinucleolar heterochromatin and nucleoli, result in changes in the structural integrity of the nucleolus. Wild type and DAXX null cells were labeled with antibodies against B23 (Fig. 4a), a protein enriched in the granular component (GC), and also found in the dense fibrillar component (DFC) of nucleoli [7, 8]. We observed a similar number of large nucleoli with a mean of 6.0 (SE 0.15) in wild type and 5.6 (SE 0.13) in DAXX null fibroblasts. However, we also observed, in some cells, between 1 and 9 very small accumulations of B23 which we refer to as “mini nucleoli” (Fig. 4a, arrowheads). Since the GC forms the outermost region of the nucleolus, B23 distribution has a ring-like pattern. Line scan analysis of intact nucleoli shows the doublet pattern of B23 intensity whereas mini nucleoli, due to their small size, appear as singlets in the line scan intensity plots. Using this feature of B23, the quantification revealed that 83 % of DAXX null cells contain one or more mini nucleolus in contrast to the 15 % of wild type cells having at least one mini nucleolus (Fig. 4b).

### Dispersed rDNA genes in the absence of DAXX

Ribosomal DNA (rDNA) genes associate with the cytogenetically discrete nucleolus organizer regions (NORs) [45]. As nucleoli form from NORs, it is assumed that rDNA is sufficient to establish a functional nucleolus [27, 38]. Since perinucleolar heterochromatin is intimately linked to rDNA gene regulation and stability [29, 49], we wanted to determine if the organization of rDNA genes was disrupted in the absence of DAXX. To test this, we performed an immuno-FISH experiment using antibodies against B23 as a marker for the nucleolus and FISH probes against rDNA genes (Fig. 4c). In wild-type cells, rDNA repeats are clustered and localized within the confines of the nucleolus as visualized by B23. In the absence of DAXX, however, we observed an increase in the fraction of rDNA foci localized outside the nucleolar boundaries (Fig. 4c, d). Taken together, these data demonstrate that DAXX-dependent heterochromatin organization and the structural integrity of the nucleolus are intimately linked. Furthermore, we conclude that the observed increase in the number of mini nucleoli is likely caused, in part, by the dispersal of rDNA genes.

### DAXX-dependent chromatin accessibility

**Fig. 5:**
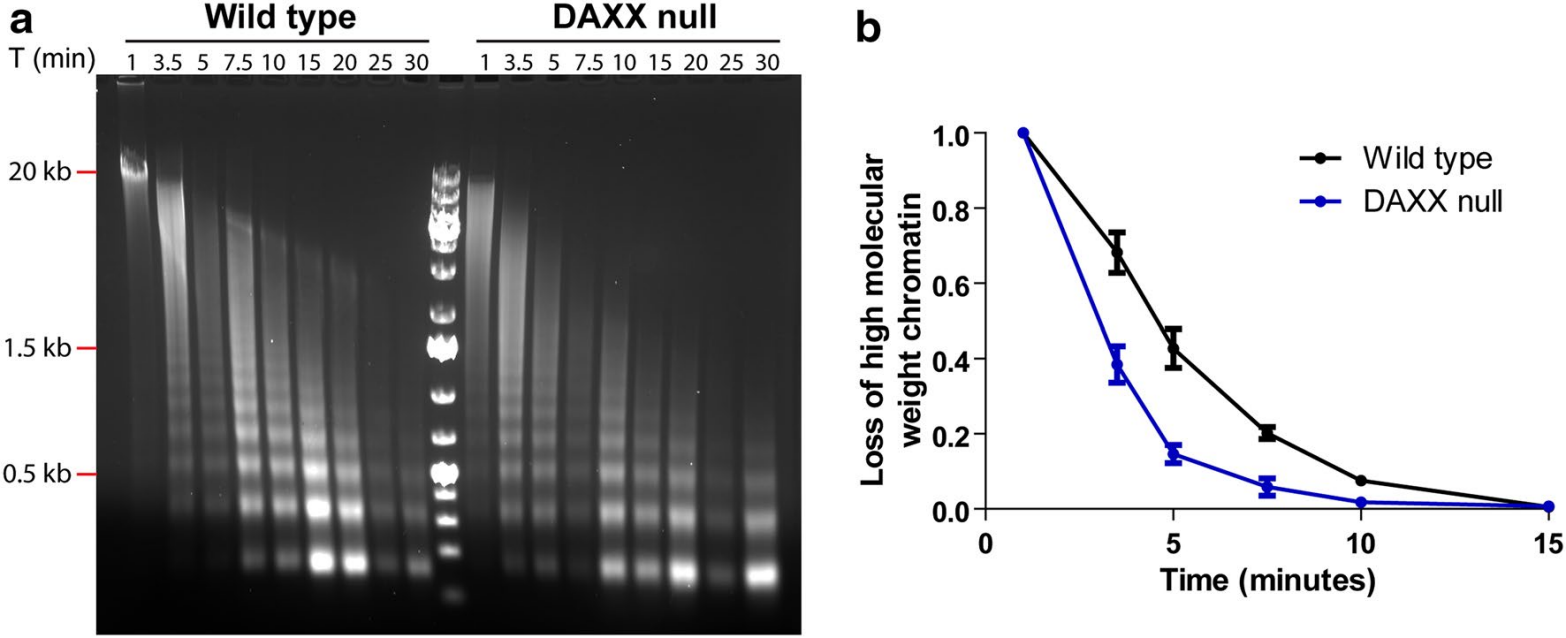
DAXX null cells are more sensitive to micrococcal nuclease digestion. **a** Micrococcal nuclease digestion of wild type and DAXX null cells. DNA was isolated from extracted nuclei after digestion for the indicated time (T) in minutes and subjected to agarose gel electrophoresis. **b** The rate of loss of high molecular weight chromatin

We showed that the loss of DAXX has consequences, at a local level, on subnuclear structures (Figs. 2, 3, 4). Next, we aimed to determine if there are global changes in chromatin structure in the absence of DAXX. To address this, nuclei from wild type and DAXX null cells were subject to micrococcal nuclease digestion and the isolated DNA was separated by agarose gel electrophoresis (Fig. 5a). From the earliest time points, DNA from DAXX null cells was significantly more sensitive to micrococcal nuclease compared to wild-type cells such that mononucleosomal DNA was evident at 3.5 min in DAXX null cells, whereas in wild-type cells, this level of digestion did not occur until 7.5 min. To quantify this observation, the rate of loss of high molecular weight chromatin was calculated by measuring the signal ≥1.5 kb and the total signal in the lane for the 3.5–15 min time points [24]. Each ratio was normalized to the 1 min time point and plotted as a time course (Fig. 5b). The rate of digestion of high molecular weight chromatin was indeed higher in the absence of DAXX, leading us to conclude that the loss of DAXX not only caused local changes in pericentric heterochromatin and rDNA organization but increased global chromatin sensitivity to micrococcal nuclease digestion.

## Discussion

The incorporation of histone variants into specific genomic regions by their corresponding chaperones and chromatin assembly pathways, together with the particular patterns of histone post-translational modifications establish heterochromatin domains required for genome integrity and the formation of cell type-specific chromatin landscapes. Using a model system that targets an H3.3-specific histone chaperone and a technique that allows us to visualize chromatin in situ, our study provides insight into the role that DAXX plays on the structural organization of pericentric heterochromatin domains in mouse cells.

### DAXX establishes heterochromatin landscapes

Here, we show that in the absence of DAXX, there are dramatic changes in the organization of H3K9me3-enriched heterochromatin domains (Figs. 1, 2). These changes include (1) the loss of typical chromocentre structure (e.g., radial symmetry); (2) loss of overlap between non-nucleolar and perinucleolar compact chromatin and H3K9me3, ectopic accumulation of compact chromatin not marked by H3K9me3; (3) increase in H3.3 associated with H3K9me3-enriched chromatin domains, and (4) loss of structural boundaries between heterochromatin and the nucleolus. The DAXX/ATRX chromatin assembly specifically deposits H3.3 into constitutive heterochromatin, including pericentric satellite repeats [16, 19, 21, 25, 47]. This deposition machinery is distinct from both the HIRA-containing complex required for H3.3 deposition in transcribed gene bodies and promoters and from the complex responsible for H3.3 enrichment at other regulatory elements [42]. It has been shown that upon CAF-1 depletion and the subsequent impairment of H3.1 incorporation, HIRA can facilitate the deposition of H3.1 into replication sites [51]. In contrast, CAF-1 was identified in H3.3-containing complexes in DAXX null MEFs [19]. As well, overexpression of the centromere-specific H3 variant CenH3 led to DAXX-mediated, rather than HJURP-mediated, deposition of CenH3 outside of centromeric chromatin [41]. Hence, one can conclude that salvage pathways exist, which compensate, at least in part, for chromatin assembly defects that occur upon loss of a specific histone chaperone [51]. But these pathways can also mistarget histone variants. Since the DAXX-deficient deposition pattern of H3.3 is not currently known, it is possible that HIRA-containing or other chromatin assembly complexes drive the deposition of H3.3 into aberrant genomic loci in the absence of DAXX, and in turn, alter the epigenetic profile of H3K9me3. The consequences of the mistargeting of H3.3 could include, but are not limited to atypical associations between H3.3 with compact chromatin, structurally disorganized domains of compact chromatin, aberrant spatial relationships of H3K9me3 with major satellite DNA, and changes to the overall heterochromatin landscape.

### The loss of DAXX causes an uncoupling of an epigenetic mark from the underlying chromatin structure

Here we show that the DAXX phenotype includes global changes in the heterochromatin landscape with the appearance of large spatial domains of compact chromatin lacking the H3K9me3 histone modification (Fig. 2). As these regions of compact chromatin are juxtaposed to H3K9me3-enriched domains, it hints towards a loss of a critical boundary such that what we are seeing could be a spreading of heterochromatin-like compaction into new domains, albeit that lack H3K9me3. The heterochromatin-like compaction may not necessarily be an induction of new heterochromatin, but could be a spatial reorganization of existing domains. A precedent for the reorganization of heterochromatic loci into spatially confined volumes is observed in chromocentre clustering during myogenesis [9, 58] or the formation of senescence-associated heterochromatin foci (SAHFs) [14, 48].

Two principles emerge from our observations of DAXX null cells. The first is that small-scale local epigenetic changes can induce global reorganization events. The second is that compact chromatin can be uncoupled from repressive histone modifications (i.e., H3K9me3). These principles have been observed in other contexts. In senescence, for example, local changes in regulation lead to massive global chromatin reorganization. SAHFs, which form after the induction of senescence, are DAPI-dense foci enriched in compact heterochromatin comprised of an H3K9me3-rich core, surrounded by a less densely packed H3K27me3 ring [14, 48]. These structures do not represent the de novo formation of heterochromatin domains since the genomic distributions of H3K9me3 and H3K27me3 remain relatively unchanged following senescence. Instead, these domains spatially relocalize within their chromosome territory, demonstrating that subtle epigenetic changes can lead to massive changes in global chromatin architecture. In regard to the second principle, the uncoupling of a heterochromatin mark and compact chromatin, chromatin compaction in senescence (SAHFs) still occurs with greatly reduced levels of H3K9/K27me3 [14]. Also, a loss of correlation of H3K9me3 and dense chromatin in mouse cells is observed in ESCs and in iPS reprogramming [23].

In contrast to senescent cells, which are more resistant to micrococcal nuclease digestion [48], DAXX null cells are more sensitive globally to nuclease digestion (Fig. 5). Hence, the large blocks of compact chromatin next to the H3K9me3-enriched heterochromatin domains may not reflect an increase in nuclease-resistant chromatin but simply the reorganization of existing domains into clusters of compact chromatin. But these new chromatin structures, though compact, may not in fact be heterochromatin or nuclease resistant; they may have the biochemical signals that lead to compaction but not those that are responsible for nuclease resistance. Such uncoupling of a morphological compact state and biochemically defined heterochromatin has been described (Even-Faitelson et al. 2015). Even though the absolute volume of compact chromatin in either wild type or DAXX null cells is not known, the relative volume does not have to differ simply because a difference in global nuclease sensitivity is observed. Our interpretation of the induction of newly observed compact domains next to pre-existing compact heterochromatin domains is that an inappropriate targeting or loss of targeting of H3.3 has led to the loss of critical boundaries maintained by DAXX. If the newly observed compact domains are not nuclease resistant, as expected for heterochromatin [26], the observed increase in nuclease sensitivity is arising in other regions of the genome. If so, the loss of proper H3.3 deposition is allowing for new (dispersed) regions that increase global nuclease sensitivity.

### DAXX maintains the structural integrity of nucleoli

The nucleolus, the most prominent structure in the nucleus and the site of ribosome biogenesis, is surrounded by blocks of condensed heterochromatin [32]. It was shown that the formation of these domains that contain rDNA and centric/pericentric regions of the genome requires the silencing of rDNA repeats [3, 29]. In this context, the nucleolus is a structural platform for the maintenance and possibly the establishment of heterochromatin domains [30]. We show here that the structural integrity of the nucleolus is compromised in the absence of DAXX (Fig. 4). The phenotype is characterized by an increase in the number of cells containing mini nucleoli as well as a dispersal of rDNA genes outside of the nucleolus. We speculate that these observations may be caused by changes in the H3K9me3-pattern of some of the rDNA chromatin. It was shown that Drosophila Su(var) mutants that have reduced levels of H3K9 methylation exhibit multiple ectopic nucleoli. While the ectopic nucleoli could have formed from dispersed nucleolar material, it is equally likely that they were caused by a mislocalization of rDNA [49]. The principal rDNA silencing pathway is NoRC (nucleolar remodeling complex), consisting of TIP5 and ATPase SNF2h [54, 57]. Depletion of TIP5 not only reduces rDNA silencing, but it also impairs the formation of perinucleolar heterochromatin [29]. It was recently discovered that PARP1 (poly(ADP-ribose)-polymerase-1) associates with TIP5 and it is involved in rDNA silencing [31]. Interestingly, PARP1 was purified from H3.1- and H3.3-containing complexes [19]. Whether the disruptions of heterochromatin result from a mislocalization of rDNA genes or changes in nucleolar integrity due to disruptions in heterochromatin formation remain to be elucidated.

### DAXX-dependent selective methylation of H3.3K9me3

**Fig. 6:**
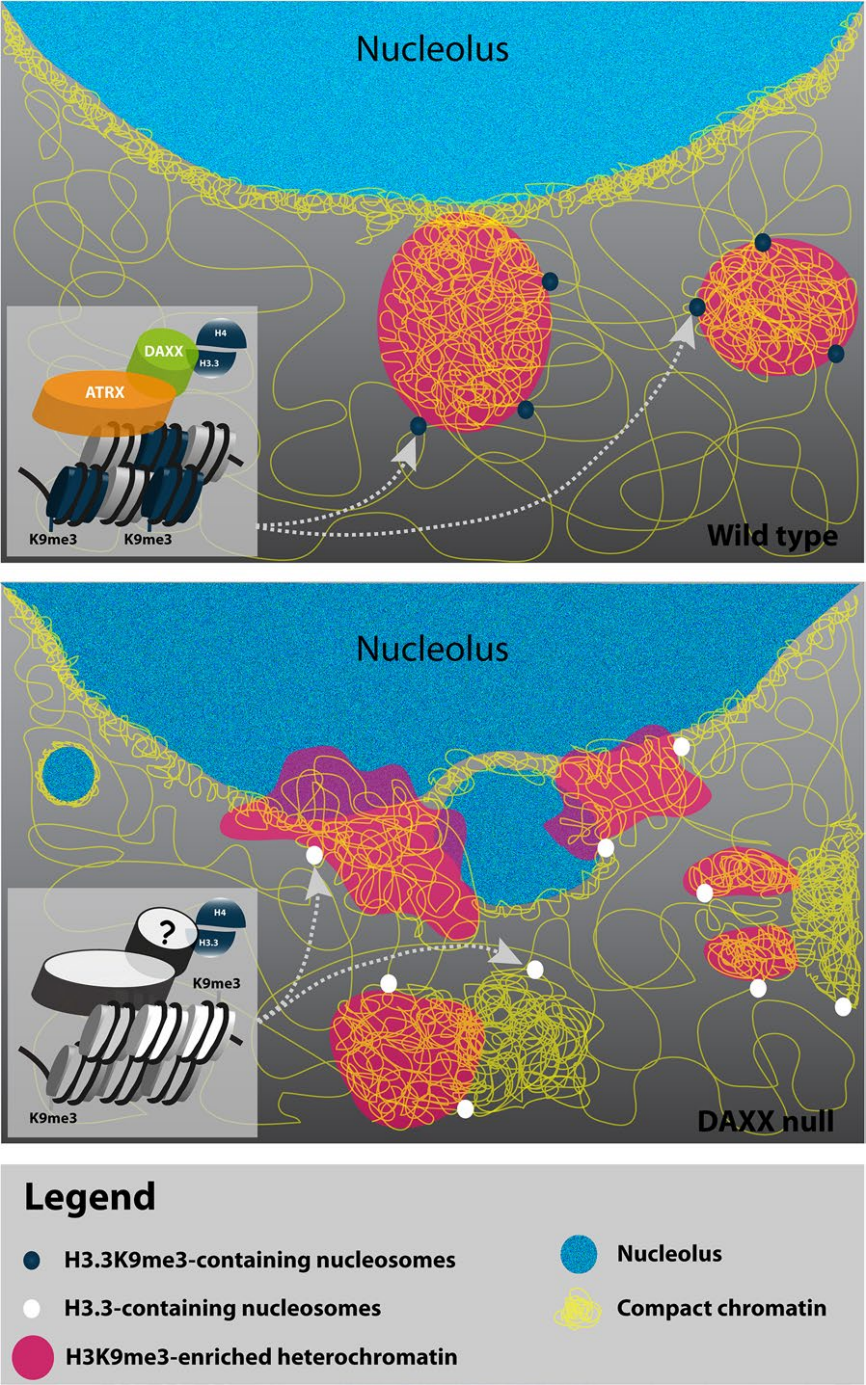
Speculative cartoon model of DAXX-dependent nuclear organization. The DAXX/ATRX complex selectively deposits H3.3 on the periphery of H3K9me3-enriched chromatin domains (*top panel*). This DAXX-dependent H3.3K9me3 epigenetic profile may index specific genomic regions, thereby marking the boundaries of and establishing pericentric constitutive heterochromatin domains (non-nucleolar and perinucleolar). In the absence of DAXX (*middle panel*), an aberrant pattern of H3.3 deposition may result in the misappropriation of heterochromatin boundaries between H3K9me3-enriched chromatin and the chromatin surrounding these regions. Consequently, ectopic domains of compact chromatin may be formed and the structural integrity of nucleoli may be compromised

We propose a model where the DAXX-dependent deposition of H3.3 plays a role in marking appropriate H3K9me3 boundaries in order to establish global constitutive heterochromatin domains (Fig. 5). This model is consistent with the ‘H3 barcode hypothesis’ which proposes that H3 variants index the genome by creating different chromosomal regions [33] and is also supported by a recent finding that identifies a DAXX-dependent H3.3K9me3 pattern important for establishing a heterochromatic state at a subset of endogenous retroviral elements in embryonic stem cells [21]. Once the variants are in place, histone PTMs would serve to regulate gene expression profiles and establish defined chromatin landscapes. Indeed evidence is emerging supporting the importance of the selective modification of histone variants. Santenard et al. [53] reported that the ectopic expression of a mutant H3.3 (H3.3K27R) impairs heterochromatin formation and development at the blastocyst stage. It was also shown that the balance between H3.3 and H1 is required for chromosome segregation and development to the blastocyst stage, and key to these processes, is the methylation of H3.3K36 [43]. It is possible that DAXX, through the deposition of H3.3 on the periphery of H3K9me3-enriched chromatin, indexes specific genomic regions and marks the boundaries of pericentric constitutive heterochromatin domains (Fig. 6). In this context, H3.3K9me3 establishes both pericentric and perinucleolar heterochromatin domains. In the absence of DAXX, the pattern of H3.3K9me3 results in the misappropriation of heterochromatin boundaries between H3K9me3-enriched chromatin and the chromatin surrounding these regions. Consequently, ectopic domains of compact chromatin lacking H3K9me3 may be formed, and changes to the epigenetic profile of rDNA (between pericentric silenced rDNA and actively transcribed rDNA) may lead to disrupted nucleoli.

### H3.3 enrichment at constitutive heterochromatin domains

While originally found accumulated at active sites of transcription, including rDNA [1], the discovery of novel chromatin assembly pathways containing the nuclear protein DAXX [19, 25] has placed H3.3 into repetitive regions of the genome including telomeres [25] and the satellite repeats of pericentric heterochromatin domains [16, 19, 47]. These findings prompted us to investigate the distribution pattern of H3.3 with respect to H3K9me3-enriched chromocentres and we found that that the percent association between H3.3 and H3K9me3-enriched chromatin domains is increased in the absence of DAXX (Fig. 1). Drane et al. [19] showed that reintroduced DAXX into MEFs localizes ectopically expressed H3.3 to PML NBs. Similarly, Corpet et al. [16] demonstrate the DAXX-dependent targeting of newly synthesized H3.3 to PML NBs in both proliferating and senescent human cells. It was later shown, again in human cells, that DAXX is targeting the soluble pool of (H3.3-H4) dimers to PML NBs pending chromatin deposition, rather than localizing H3.3-incorporated chromatin to PML NBs [17]. While these studies make use of imaging techniques and show H3.3 at PML NBs, H3.3 at pericentric heterochromatin has only been demonstrated using ChIP-based methods [16, 19, 47]. A drawback of such biochemistry-based techniques in the context of pericentric heterochromatin domains (chromocentres), however, is that chromocentres are studied as a population and with that comes the assumption that chromocentres are homogeneous. We show, however, that H3.3 enrichment occurs at a fraction of chromocentres, implying that chromocentres are heterogeneous. This is expected since the number of chromocentres is highly variable within cells of a given cell type as well as between different cell types [13, 44]. Importantly, we show that H3.3 is not found in the core of H3K9me3-enriched chromatin domains, but rather associates with the periphery (Additional file 2). Therefore, the enrichment of H3.3 with major satellite repeat DNA might be due to the 3D arrangement of DAXX-dependent H3.3 target loci with pericentric chromatin, and not necessarily the direct deposit of H3.3 into these specific repeats. Therefore, the question remains as to which specific genomic regions DAXX is targeting H3.3 and what specifies the targeting.

### The disparate functions of DAXX

Although originally identified in a screen designed to identify proteins that could bind to the cytoplasmic domain of the Fas receptor [61], DAXX is predominantly a nuclear protein which associates with both promyelocytic leukemia nuclear bodies (PML NBs) and ATRX-containing heterochromatic regions in S-phase of the cell cycle [36, 37]. The targeted deletion of the *Daxx* gene results in embryonic lethality characterized by the loss of identifiable tissue types [46]. This observation is not surprising since global chromatin organization, including the amount and distribution of compact chromatin domains, is cell-type specific [50]. We propose that DAXX, via the deposition of H3.3, could function in establishing the organization of heterochromatin domains required for tissue differentiation in the developing mouse embryo.

## Conclusions

We identify a novel role of DAXX as a major regulator of subnuclear organization through the maintenance of the global heterochromatin structural landscape. We also show a direct link between DAXX-dependent H3.3 deposition and the structural integrity of the nucleolus. In addition, we demonstrate the severe in vivo consequences that the loss of a histone chaperone can have on global nuclear organization.

## Methods

### Cell culture and transfection

T-immortalized wild type (DAXX^+/+^) and DAXX null (DAXX^−/−^) mouse embryonic fibroblasts (MEFs) [37] were cultured standard culture media. For the transfection experiment, cells were transiently transfected (Lipofectamine 2000 Transfection Reagent, Invitrogen) with a FLAG-HA-H3.3 plasmid [47].

### Immunofluorescence microscopy

Cells were fixed in 2 % paraformaldehyde (Electron Microscopy Sciences) for 10 min at room temperature (RT) and permeabilized in 0.5 % Triton X-100 (BioShop) for 5 min at RT. The primary antibodies used were rabbit anti-H3.3 (Abcam), rabbit anti-H3K9me3 (gift from P Singh), mouse anti-H3K9me3 and mouse anti-H4K20me3 (H Kimura), mouse anti-B23 (Santa Cruz Biotechnology), rabbit anti-nucleolin (Santa Cruz Biotechnology), and mouse anti-FLAG (Sigma-Aldrich). The secondary antibodies used were rabbit or mouse Cy2, Cy3, and Cy5 (Jackson ImmunoResearch Laboratories). Cells were soaked in DAPI and mounted in buffered glycerol with 4 % *n*-propyl gallate.

Images were collected on an Olympus IX81 inverted microscope equipped with a Cascade II CCD camera (Photometrics) using either a 60× or 100× oil-immersion objective lenses. MetaMorph Microscopy Automation & Image Analysis Software (Molecular Devices) was used to collect images. Images were processed with Volocity 3D Image Analysis Software (PerkinElmer) and Photoshop (Adobe). Graphs and statistics were constructed using GraphPad Prism (GraphPad Software Inc) and reported as standard error of the mean. Line scan data was collected using ImageJ (National Institute of Health) and the histograms constructed using GraphPad Prism.

### Correlative LM/ESI microscopy

For a detailed sample preparation protocol and ESI procedure, see Ahmed et al. [2]. Briefly, cells were immunolabeled and post-fixed in 1 % glutaraldehyde (Electron Microscopy Sciences). Immunogold labeling was performed with Nanogold reagents (Nanoprobes). Following dehydration, cells were embedded in Quetol 651 resin (EMS). Samples were sectioned to 70 nm by an ultramicrotome (Leica). Grids containing the sample sections were imaged on a fluorescent microscope (Leica). Following carbon coating, electron micrographs were collected on a transmission electron microscope (Tecnai 20, FEI). To generate the phosphorus and nitrogen images, the microscope was operated at 200 kV using a post column filter (Gatan) at 120 and 155, and 385 and 415 eV, respectively. ESI images were generated as previously described [2]. In the overlay images, yellow represents nucleic acid-based structures, and cyan represents protein-based structures. To correlate the fluorescence signal with the underlying chromatin structure, the fluorescence images were overlaid onto the phosphorus-enhanced low magnification electron micrographs and the H3K9me3-positive regions identified and imaged. For presentation, the approximate boundaries of the H3K9me3 region are marked by a dotted line. Images were processed with Photoshop (Adobe).

### 3D FISH

Briefly, cells grown on glass slides were immunolabeled for H3K9me3 or B23, post-fixed in 2 % paraformaldehyde, washed in PBS, and maintained overnight in 20 % glycerol in PBS at 4 °C. Cells were snap frozen, partially thawed, and then returned to the 20 % glycerol solution (repeated 5 times). Slides were washed in PBS, treated with 0.1 N HCl for 5 min, washed in 2× SSC, and stored overnight in a solution of 50 % formamide in 2× SSC at 4 °C. Slides were denatured and hybridized overnight at 37 °C with major satellite and rDNA FISH probes (The Centre for Applied Genomics) directly labeled with spectrum orange fluor-conjugated nucleotides. Slides were mounted using Vectashield (Vector Laboratories) containing DAPI [15].

### Western blot

Harvested cells were resuspended with 9 M urea in 10 mM Tris–Cl, pH 6.8. Samples were quantified using the Bio-Rad Protein Concentration Assay (Bio-Rad Laboratories). Protein samples were resolved on SDS-PAGE gels and transferred onto nitrocellulose membranes (GE Healthcare). Membranes were blocked overnight in 5 % skim milk powder in TBST (0.05 % Tween-20, Bio-Rad). Primary antibodies used were mouse anti-H3K9me3 (gift from H. Kimura), rabbit anti-H3.3 (Abcam), and rabbit anti-GAPDH (Sigma). Secondary antibodies used were mouse and rabbit anti-HRP (Sigma). Detection was performed using the Western Lightning Plus ECL system (PerkinElmer).

### Micrococcal nuclease assay

Harvested wild type or DAXX null MEFs were resuspended in nuclear extraction buffer A (85 mM KCl, 10 mM Tris pH 7.5, 0.2 mM spermidine, 0.2 mM EDTA, 160 mM sucrose, 250 μM PMSF), placed on ice for 5 min, and lysed in an equal volume of nuclear extraction buffer B (buffer A supplemented with 0.1 % NP40) on ice for 2 min. Nuclei were washed and resuspended in digestion buffer (50 mM Tris pH 7.5, 20 mM KCl, 0.32 M sucrose, 4 mM MgCl_2_, 3 mM CaCl_2_) and digested with micrococcal nuclease (10 units/ml) for the indicated times. The reactions were stopped using an equal volume of stop buffer (2 % SDS, 200 μg/ml PK, 10 mM EDTA). The DNA was purified by phenol–chloroform extraction and ethanol precipitation and subject to agarose gel electrophoresis. The volume of high molecular weight DNA was quantified using ImageJ and normalized to the 1 min time point. The graph was generated using GraphPad Prism.

## Additional files

10.1186/s13072-015-0036-2 Localization of endogenous DAXX.
10.1186/s13072-015-0036-2 Peripheral association of H3.3 with H3K9me3-enriched domains.
10.1186/s13072-015-0036-2 Distribution of H3K9me3, H4K20me3, and HP1 in wild type and DAXX null fibroblasts.

## Abbreviations

DAXX: death domain-associated protein
H3.3: replication-independent H3 variant H3.3
LM/ESI: correlative light microscopy electron spectroscopic imaging
MEF: mouse embryonic fibroblast
rDNA: ribosomal DNA
