# Supplementary material for: The histone chaperone DAXX maintains the structural organization of heterochromatin domains
Source: Epigenetics Chromatin. 2015 Oct 21;8:44. doi: 10.1186/s13072-015-0036-2 (PMC4617904; doi:10.1186/s13072-015-0036-2)
Supplement: Supplementary file 1 — 10.1186/s13072-015-0036-2 Localization of endogenous DAXX. [file 13072_2015_36_MOESM1_ESM.pdf]

Additional files

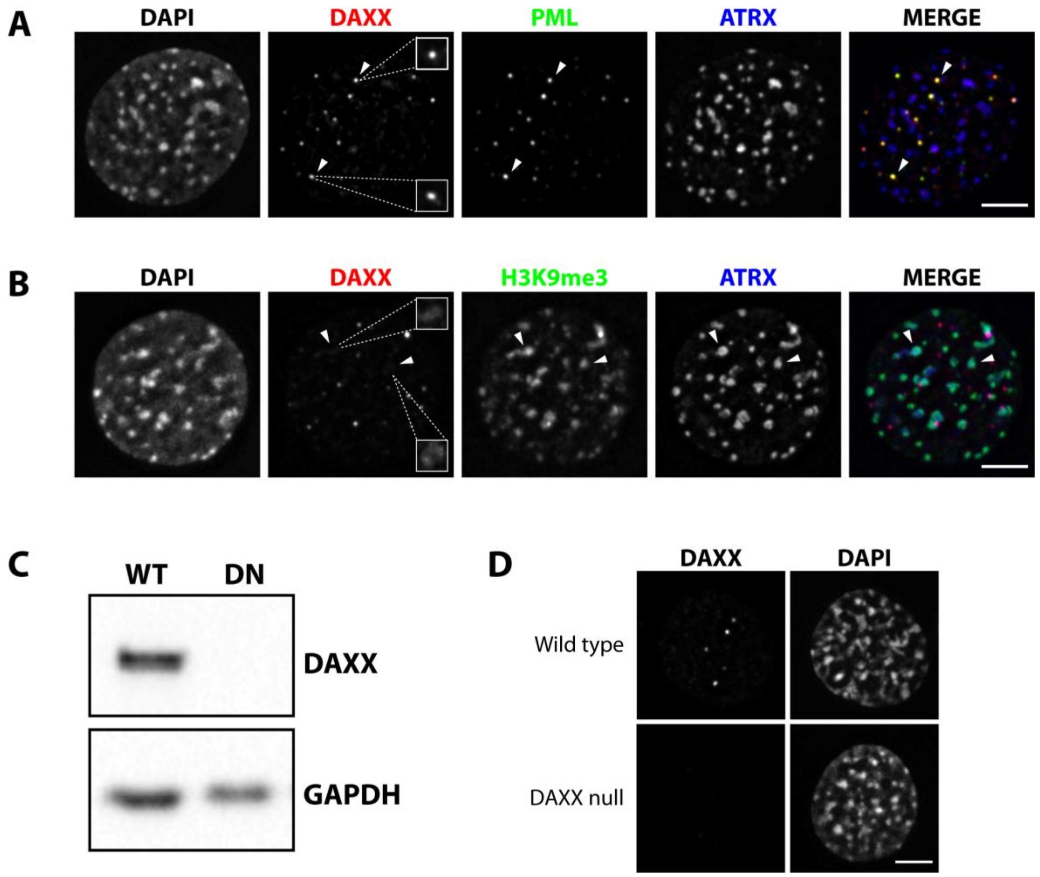

Additional file 1: Localization of endogenous DAXX.

(A) Wild type cells immunolabeled for DAXX (red), PML (green), and ATRX (blue). Arrowheads and enlarged regions indicate representative DAXX-containing PML NBs. Scale bar, 5 µm. (B) Wild type cells immunolabeled for DAXX (red), H3K9me3 (green), and ATRX (blue). Arrowheads and enlarged regions indicate DAXX-enriched chromocentres. DAXX levels in the enlarged regions were contrast enhanced in Photoshop. Scale bar, 5 µm. (C) Western blot analysis of whole cell lysates from wild type and DAXX null fibroblasts. GAPDH was used a gel loading control. (D) Wild type and DAXX null cells immunolabeled with DAXX antibodies. DAXX protein is not detectable in the null cells. Scale bar, 5 µm.
