# Supplementary material for: The histone chaperone DAXX maintains the structural organization of heterochromatin domains
Source: Epigenetics Chromatin. 2015 Oct 21;8:44. doi: 10.1186/s13072-015-0036-2 (PMC4617904; doi:10.1186/s13072-015-0036-2)
Supplement: Supplementary file 2 — 10.1186/s13072-015-0036-2 Peripheral association of H3.3 with H3K9me3-enriched domains. [file 13072_2015_36_MOESM2_ESM.pdf]

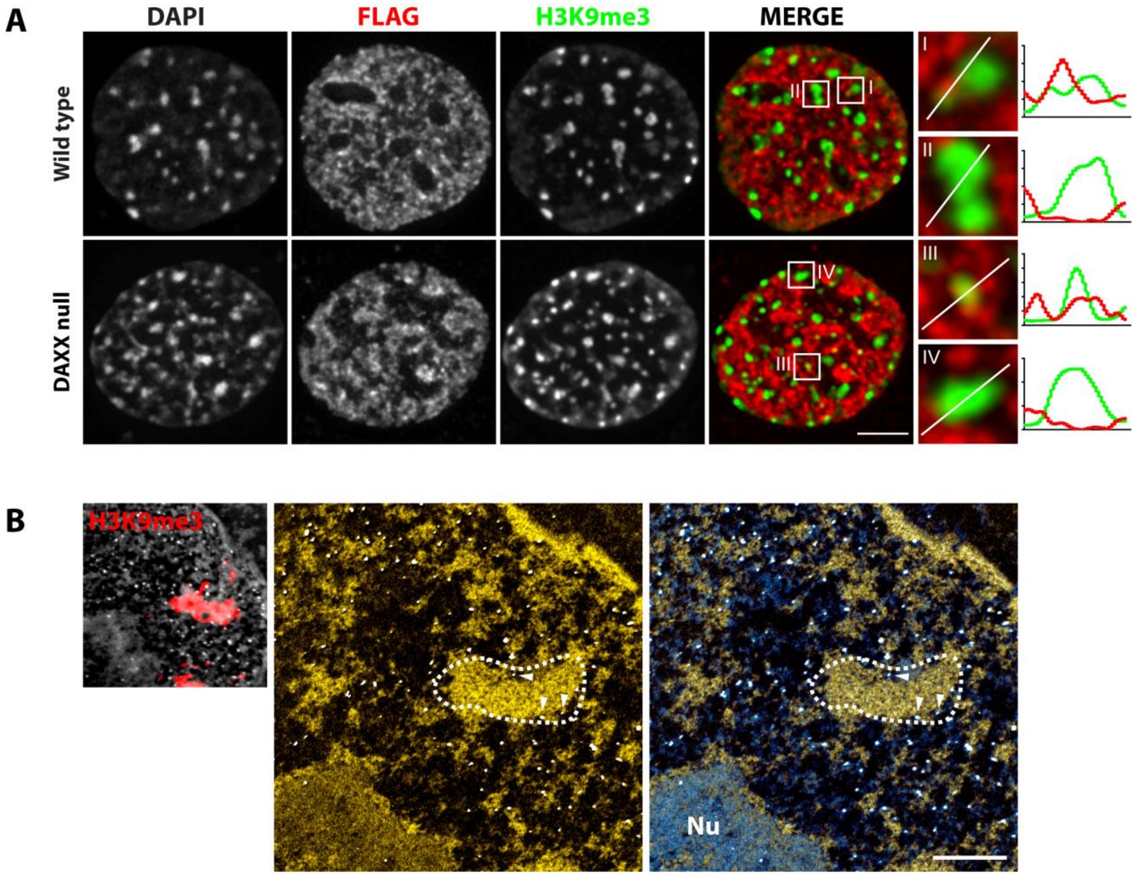

Additional file 2: Peripheral association of H3.3 with H3K9me3-enriched domains.

(A) FLAG-H3.3 transfected wild type and DAXX null fibroblasts and immunolabeled for FLAG (red) and H3K9me3 (green). Two independent line scan intensity plots are shown for each enlarged chromocentre. H3.3 associated (I, III) and non-associated (II, IV) chromocentres are shown. Scale bar, 5 µm. (B) H3K9me3 fluorescence (red) overlayed onto the mass sensitive image generating the correlative image (left panel). Electron spectroscopic imagining (ESI) for phosphorus (centre) delineates chromatin (yellow). The phosphorus image overlayed onto the nitrogen minus phosphorus micrograph delineate chromatin (yellow) and protein-based structures (cyan). Approximate boundaries of the H3K9me3-defined region are indicated by a dashed line. Small bright, white objects at the periphery of H3K9me3-enriched chromatin and in the euchromatic space represent gold particles labeling H3.3 (arrowheads). Nu, nucleolus. Scale bar, 1.0 µm.
