# Supplementary material for: The histone chaperone DAXX maintains the structural organization of heterochromatin domains
Source: Epigenetics Chromatin. 2015 Oct 21;8:44. doi: 10.1186/s13072-015-0036-2 (PMC4617904; doi:10.1186/s13072-015-0036-2)
Supplement: Supplementary file 3 — 10.1186/s13072-015-0036-2 Distribution of H3K9me3, H4K20me3, and HP1 in wild type and DAXX null fibroblasts. [file 13072_2015_36_MOESM3_ESM.pdf]

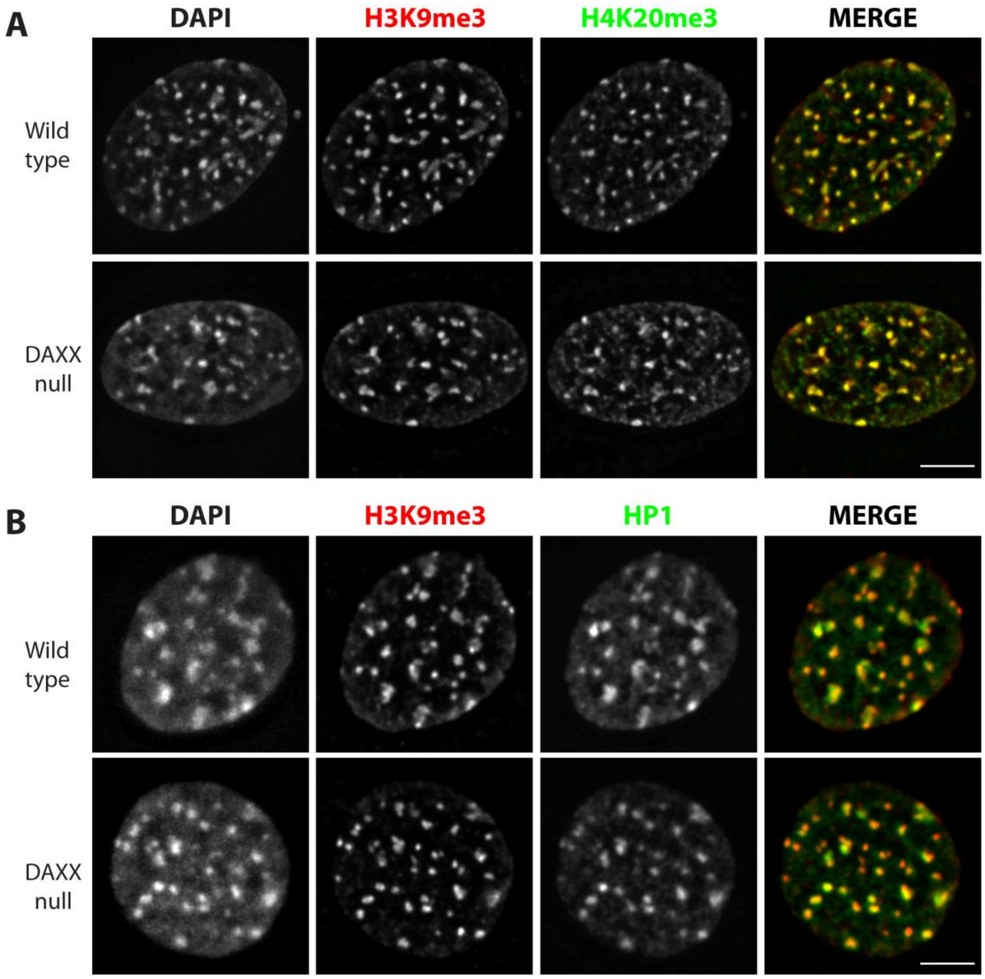

Additional file 3: Distribution of H3K9me3, H4K20me3, and HP1 in wild type and DAXX null fibroblasts.

(A) IF of wild type and DAXX null fibroblasts labeled with antibodies against H3K9me3 (red) and H4K20me3 (green). Scale bar, 5 µm. (B) IF of wild type and DAXX null fibroblasts labeled with antibodies against H3K9me3 (red) and HP1 (green). Scale bar, 5 µm.
